# Bioactivity and Molecular Responses of Bitter Gourd Leaf Extracts Against the Invasive Leafminer, *Liriomyza trifolii* (Diptera: Agromyzidae)

**DOI:** 10.3390/insects17080827

**Published:** 2026-08-10

**Authors:** Ya-Wen Chang, Ling Zhong, Zhen Yuan, Yu-Zhou Du

**Affiliations:** 1 College of Plant Protection & Institute of Applied Entomology, Yangzhou University, Yangzhou 225009, China; changyawen@yzu.edu.cn (Y.-W.C.);; 2 Jiangsu Province Engineering Research Center of Green Pesticides, Yangzhou University, Yangzhou 225009, China

**Keywords:** *Liriomyza trifolii*, *Momordica charantia*, botanical insecticide, transcriptomics, metabolomics, integrated analysis

## Abstract

*Liriomyza trifolii* is a global pest of vegetables and ornamental plants and a major invasive species in China. Long-term irrational use of chemical insecticides has reduced control efficacy, driving interest in plant-derived alternatives. Bitter gourd, *Momordica charantia*, extracts show potential against various pests, but their effects on *L. trifolii* are unknown. To address this, host suitability tests were first conducted and revealed that *L. trifolii* exhibited significantly higher adaptability to kidney bean than to bitter gourd in terms of both oviposition and feeding, and failed to complete its life cycle on bitter gourd. Further treatment with ethanol extracts of bitter gourd leaves demonstrated dose-dependent adulticidal activity; at the LC_50_ concentration, the extract not only reduced feeding punctures and oviposition, but also significantly decreased egg hatching and larval survival, while showing no significant impact on pupation or emergence. To elucidate the underlying molecular mechanisms, integrative transcriptomic and metabolomic analyses were subsequently performed. Transcriptomics identified 254 differentially expressed genes (DEGs) enriched in ribosome, oxidative phosphorylation, and peroxisome pathways, and upregulated DEGs included a vitellin-degrading protease and cytochrome P450. Meanwhile, metabolomics detected 272 differential metabolites (DEMs): upregulated metabolites were linked to purine metabolism, while downregulated ones were associated with cysteine/methionine and arginine/proline metabolism. Integrated analysis revealed “Biosynthesis of amino acids” as the sole common pathway, with S-adenosyl-L-homocysteine, *N*-succinyl-LL-2,6-diaminoheptanedioate, and glutamine synthetase (*Ltr05G008090*) showing significant correlations. These findings suggest that reprogramming of amino acid and nitrogen metabolism may participate in the response and could serve as a candidate response pathway. Accordingly, this study may offer a theoretical basis for the future development of botanical insecticides based on bitter gourd leaf extract against *L. trifolii*.

## Simple Summary

*Liriomyza trifolii* is recognized as a destructive invasive pest affecting vegetable and ornamental crops worldwide, including China, where prolonged chemical insecticide use has led to reduced efficacy. In this study, the potential of bitter gourd (*Momordica charantia*) leaf extract as a botanical alternative was investigated. Host suitability tests demonstrated that *L. trifolii* exhibited significant oviposition and feeding adaptability for kidney bean over bitter gourd, with complete life cycle failure observed on the latter. Application of ethanol extracts resulted in dose-dependent adulticidal activity. At the LC_50_ concentration, feeding punctures and oviposition were notably reduced, and egg hatching and larval survival were significantly decreased, whereas pupation and emergence were not markedly affected. Integrative transcriptomic and metabolomic analyses were subsequently performed to elucidate underlying mechanisms, revealing 254 differentially expressed genes and 272 differential metabolites. Joint pathway analysis identified “Biosynthesis of amino acids” as the sole common pathway, with notable associations involving S-adenosyl-L-homocysteine, *N*-succinyl-LL-2,6-diaminoheptanedioate, and glutamine synthetase. These findings suggest that the reprogramming of amino acid and nitrogen metabolism may participate in the treatment response and could serve as a candidate response pathway. As such, this pathway may provide a theoretical basis for future development of plant-derived insecticides against *L. trifolii*.

## 1. Introduction

*Liriomyza trifolii* (Burgess) is a worldwide pest of vegetables and ornamental plants, and also an important invasive alien species in China [1]. Both larvae and adults of this pest cause direct damage: larvae mine within host leaves, forming serpentine galleries that inhibit photosynthesis, while adult feeding and oviposition punctures create entry points for pathogen infection [2,3]. In recent years, with the large-scale expansion of facility agriculture, the damage caused by leafminer pests has become increasingly severe, gradually rendering them key insect pests in vegetable production. This species has a broad host range, with crops in the families Fabaceae, Solanaceae, Cucurbitaceae, and Brassicaceae being the most severely affected [2,4,5]. Furthermore, *L. trifolii* is characterized by its small body size, short developmental period, high fecundity, rapid dispersal ability, and widespread generational overlap, all of which contribute to its high risk of outbreak in the field [6].

For a long time, the control of *L. trifolii* has relied primarily on chemical insecticides. However, due to its high levels of resistance to multiple conventional insecticides, the efficacy of traditional chemical control has been steadily declining, accompanied by serious issues such as pesticide residues and environmental pollution [7,8]. In this context, the development of environmentally friendly control strategies based on the behavioral regulation of insects has become an important direction for integrated pest management of this species [6,9]. As a key component of green pest management, plant-derived bioactive substances can interfere with pest feeding, growth, development, and reproduction through the antifeedant, repellent, or toxic effects of secondary metabolites, thereby effectively suppressing field populations [10]. Compared with chemical pesticides, botanical pesticides offer several advantages, including good environmental compatibility, low residue levels, low risk of resistance development, wide availability, and compatibility with other agents, making them important tools in sustainable pest management [11,12,13,14].

Bitter gourd (*Momordica charantia* L.) is an annual climbing herb in the family Cucurbitaceae, genus Momordica. Its fruits are well known for their characteristic bitter taste and are widely used as both a vegetable and a traditional medicinal ingredient in many regions, particularly in Asia, Africa, and the Caribbean [15]. Beyond its nutritional and medicinal values, bitter gourd also exhibits significant pest repellent and resistance properties in agroecosystems, positioning it as a highly promising eco-friendly resource for crop protection [16]. Previous studies have demonstrated that bitter gourd leaf ethanol extracts effectively kill larvae of the dengue vector *Aedes aegypti* [17]; and the trypsin inhibitor II present in its seeds suppresses the development of the cotton bollworm [18]. In the context of leafminer management, bitter gourd has also shown notable application potential: its volatile organic compounds exert strong olfactory repellent effects on adult *L. sativae*, interfering with host location and colonization [19]; its leaf ethanol extracts and ethyl acetate fractions exhibit significant antifeedant and oviposition-deterrent activities, effectively reducing the population density of subsequent generations [19,20]. Further studies have revealed that the resistance of bitter gourd to *L. sativae* stems from its enriched secondary metabolites, among which cucurbitacin B serves as a key active component. This compound efficiently inhibits egg hatching and blocks the growth and development of neonates, preventing larvae from completing their life cycle within leaf tissues [21]. These physical defenses, chemical defenses, and behavioral interference together constitute the multi-layered resistance system of bitter gourd.

Notably, most of the previous studies on the insecticidal activity of bitter gourd have focused exclusively on *L. sativae*. With respect to *L. trifolii*, only Mekuria et al. [22,23] have identified cucurbitane glycosides and triterpenoids from bitter gourd extracts and demonstrated that their application to kidney bean leaves exerted significant oviposition-deterrent effects on adult *L. trifolii*; however, neither study validated the insecticidal bioactivity nor elucidated the underlying molecular mechanisms. Nevertheless, accumulating evidence indicates that *L. trifolii* exhibits significantly greater competitive advantages over *L. sativae* in terms of resource utilization, environmental adaptability, and interspecific competition [24,25,26]. Given that both species belong to the same genus and share highly similar biological traits and damage patterns, it is reasonable to hypothesize that the secondary metabolites of bitter gourd possess comparable control potential against *L. trifolii*. To address this, the present study employs bitter gourd as the experimental material, prepares leaf extracts via ultrasonic-assisted extraction combined with rotary evaporation concentration, and systematically evaluates their effects on the growth and development of *L. trifolii* across various life stages. Concurrently, integrated transcriptomic and metabolomic analyses are employed to investigate, at both the gene expression and metabolite accumulation levels, the molecular regulatory networks through which bitter gourd extracts interfere with the nutritional metabolism and detoxification pathways of the target pest. The ultimate goal is to precisely identify the key metabolic pathways and functional genes involved in its anti-insect activity, thereby providing a multi-omics theoretical foundation for the development of novel green botanical insecticides specifically targeting *L. trifolii*.

## 2. Materials and Methods

### 2.1. Insects and Plants

The *L. trifolii* used in this study were originally collected from Yangzhou, Jiangsu Province (32.39° N, 119.42° E) in 2015 and subsequently reared in the laboratory on kidney bean plants (*Phaseolus vulgaris*) for multiple generations following the method of Chen & Kang [27]. Rearing conditions were as follows: temperature 25 ± 1 °C, relative humidity 60–70%, and a photoperiod of 16:8 h (L:D).

Both bitter gourd and kidney bean plants used in the experiments were grown in the laboratory. Plump, disease-free seeds of each species were selected, germinated, sown in plastic flower pots, and cultivated in a glass greenhouse with ample sunlight at 25 ± 5 °C. No pesticides or chemical fertilizers were applied throughout the experimental period, and only leaves were used for subsequent assays.

### 2.2. Comparison of Feeding and Oviposition of L. trifolii on Different Host Plants

Bitter gourd and kidney bean seedlings at the two-true-leaf stage, with uniform growth and free of pests, were selected for the experiment. The seedlings were individually transplanted into 1000 mL plastic cups, with one plant per cup. All plants were maintained under the same light, temperature, and water conditions to minimize interference from environmental factors. Newly emerged unmated adult *L. trifolii* were introduced into the plastic cups containing bitter gourd or kidney bean plants at a density of two pairs per cup. The cup openings were covered with gauze to prevent adult escape while ensuring adequate air circulation. At 48 h post-infestation, the adults were carefully aspirated from the cups using an aspirator, taking care not to disturb the eggs or larvae on the plants. The number of feeding punctures and eggs on each plant was observed and recorded under a microscope, and the feeding puncture and oviposition amounts were compared between bitter gourd and kidney bean. Each treatment was replicated four times.

### 2.3. Determination of the Mortality Effect of Bitter Gourd Leaf Extract on L. trifolii

Bitter gourd leaves were air-dried until brittle, then pulverized with a grinder and passed through a 40-mesh sieve to obtain dry powder. Ten grams of the powder were placed in a 500 mL conical flask, and 100 mL of 95% ethanol was added as the solvent. The mixture was placed in an ultrasonic cleaner and extracted with oscillation at 60 °C for 30 min. The extract was then filtered under vacuum, and the residue was extracted three additional times, each with 100 mL of the same solvent. All filtrates were collected and combined, then concentrated to dryness under reduced pressure using a rotary evaporator to obtain the crude extract. The extract was dissolved in ethanol with ultrasonic assistance and made up to 10 mL (equivalent to 1 g of leaf powder per 1 mL of solution). This solution was then diluted 10-fold with distilled water containing 1% Tween-80 to prepare a stock solution (100 mg/mL), which was subsequently diluted with distilled water containing 1% Tween-80 to concentrations of 1, 3, 5, 10, and 20 mg/mL of bitter gourd extract. The control group received the same solvent without the extract (distilled water containing 1% Tween-80 and the same amount of 95% ethanol). One hundred *L. trifolii* adults were used for each concentration treatment, with 20 individuals placed into each 10 mL glass tube, the openings of which were sealed with cotton. Each concentration included five biological replicates. Different concentrations of the extract were dripped onto the cotton for the insects to feed on. Mortality was checked at 24 h, and the mortality rate and corrected mortality rate were calculated. The goodness-of-fit test for each probit analysis model was evaluated using SPSS v. 16.0 (SPSS, Chicago, IL, USA), with the chi-square value and its associated *p*-value assessed (*p* > 0.05 was considered indicative of a good fit). Linear regression analysis was performed between log concentration (X) and probit (Y), and the lethal concentration (LC_50_) values along with their 95% confidence intervals were calculated accordingly.

### 2.4. Effects of Bitter Gourd Leaf Extract on the Growth and Development of L. trifolii

Kidney bean seedlings at the cotyledon stage, with uniform growth and free of pests, were selected and placed into clean 1000 mL plastic cups. The treatment group was treated with the LC_50_ concentration (feeding bioassay) of bitter gourd extract, while the control group received the same liquid formulation without the extract. The corresponding liquids were poured into Petri dishes, and the cotyledons of the kidney bean seedlings were immersed for 10 s, then removed and air-dried naturally.

Two pairs of newly emerged unmated male and female *L. trifolii* adults were introduced into each cup, and the cup openings were sealed with gauze. The insects were reared in an indoor environment at 25 ± 1 °C, 60–70% relative humidity, and a photoperiod of 16:8 h (L:D). The adults were allowed to oviposit freely in the cups; all adults were removed after 48 h. The bean seedlings bearing eggs were retained and continuously cultured under the same climatic conditions. The number of feeding punctures and eggs was regularly observed and recorded. The following parameters were then calculated: egg hatching rate (number of hatched larvae/total number of eggs × 100%), larval survival rate (number of pupae/initial number of larvae × 100%), pupation rate (number of pupae/initial number of larvae × 100%), and emergence rate (number of emerged adults/number of pupae × 100%). Each treatment was replicated six times.

### 2.5. Transcriptomic and Metabolomic Analysis of L. trifolii Under Bitter Gourd Leaf Extract Treatment

Transcriptomic and metabolomic samples were from adults surviving 24 h of LC_50_ bitter gourd extract treatment. One-day-old unmated male and female adults were selected at a 1:1 ratio for the treatment. Surviving adults were then collected, with 150 adults per replicate for transcriptomic sequencing and 200 adults per replicate for metabolomic profiling, each with three biological replicates (*n* = 3), frozen in liquid nitrogen, and stored at −80 °C.

Total RNA was extracted using the SV Total RNA Isolation System kit (Promega Corporation, Madison, WI, USA). RNA quality was assessed for concentration (NanoDrop 2000) (Thermo Fisher Scientific, Wilmington, DE, USA) and integrity (Agilent 2100 (Agilent Technologies, Santa Clara, CA, USA) and LabChip GX (PerkinElmer, Waltham, MA, USA)). Libraries were constructed via oligo(dT) mRNA enrichment, fragmentation, cDNA synthesis, end repair, A-tailing, adapter ligation, AMPure XP (Beckman Coulter, Brea, CA, USA) selection, and PCR, then sequenced on an Illumina HiSeq 2500 (PE150) (Illumina, San Diego, CA, USA) by Biomarker Technologies (Beijing, China). RNA-seq data were deposited in the Sequence Read Archives (accession no. PRJNA1481800) at the National Center for Biotechnology Information (NCBI) (https://www.ncbi.nlm.nih.gov/, accessed on 2 July 2026). Raw reads were filtered (removing low-quality, adapter-containing, or >10% N reads), and clean reads were aligned to the *L. trifolii* reference genome [28] using HISAT2 (v2.0.4). Transcript assembly was performed with StringTie (v2.2.1). Gene boundaries were refined by extending UTRs, and novel transcripts/genes were identified by comparing assembled reads to existing annotations. Novel sequences were annotated against NR, Swiss-Prot, GO, COG, KOG, eggNOG, Pfam, and KEGG. RNA-seq data consist of reads derived from expressed transcripts, with a higher number of reads mapped to a specific transcript generally indicating greater abundance. However, as raw read counts are affected by transcript length and sequencing depth, normalization is essential to ensure accurate comparisons across samples. In this study, transcript and gene expression levels were quantified with the maximum-flow algorithm and normalized as FPKM (Fragments Per Kilobase of transcript per Million fragments mapped), and DEGs were identified with DESeq2 (v1.30.1), with a threshold of |fold change| ≥ 1 and *p* < 0.05. The false discovery rate (FDR), derived from *p*-value adjustment for multiple testing, was applied to assess the statistical significance of these differences. For RT-qPCR validation, eight DEGs were randomly selected; primers were designed with *Actin* as the reference [29] (Table S1). cDNA was synthesized (PrimeScript RT kit (TaKaRa Biotechnology, Dalian, China), 500 ng RNA), and qPCR was run using ChamQ Universal SYBR qPCR Master Mix (Vazyme, Nanjing, China) (40 cycles of 94 °C for 30 s and annealing at 59 °C for 30 s).

For metabolomics analysis, an LC/MS system consisting of a Waters Acquity I-Class PLUS (Waters Corporation, Milford, MA, USA) ultra-high performance liquid chromatograph coupled to a Waters Xevo G2-XS QTof (Waters Corporation, Milford, MA, USA) high-resolution mass spectrometer was employed, equipped with a Waters Acquity UPLC HSS T3 column (Waters Corporation, Milford, MA, USA) (1.8 μm, 2.1 × 100 mm). The mobile phases were 0.1% formic acid in water (A) and 0.1% formic acid in acetonitrile (B) for both positive and negative ion modes, with an injection volume of 2 μL. Under the control of MassLynx V4.2 software, the mass spectrometer operated in MSe mode to acquire both MS and MS/MS data within each cycle, simultaneously performing low-energy (off) and high-energy (10–40 V) scans at a frequency of 0.2 s per spectrum. ESI source parameters were set as follows: capillary voltage 2500 V (positive) or −2000 V (negative), cone voltage 30 V, ion source temperature 100 °C, desolvation temperature 500 °C, cone gas flow 50 L/h, and desolvation gas flow 800 L/h. Raw data were processed with Progenesis QI (v3.1) software for peak picking, alignment, and compound identification against the METLIN online database and an in-house library. After total peak area normalization, principal component analysis and Spearman correlation analysis were used to assess sample repeatability and quality control performance (Figures S1 and S2). Identified compounds were annotated with KEGG, HMDB, and Lipidmaps databases. Differential metabolites were screened using VIP scores derived from OPLS-DA modeling (ropls R package (v3.6.1), validated by 200 permutation tests) as the primary filter, followed by refinement with univariate criteria. Metabolites with VIP ≥ 1 (indicating strong contribution to group discrimination in the OPLS-DA model) were initially selected for groups with biological replicates (Figure S3). These candidates were further filtered using |fold change| ≥ 2 (encompassing both FC > 2 for up-regulation and FC < 1/2 for down-regulation) and *t*-test *p*-value < 0.05 to ensure statistical robustness. KEGG pathway enrichment of differential metabolites was evaluated using the hypergeometric distribution test. Metabolomics data have been deposited in the Science Data Bank (ScienceDB) and are accessible at https://doi.org/10.57760/sciencedb.41966.

Integrated analysis of DEGs and DEMs was performed on BMKCloud (www.biocloud.net, accessed on 1 March 2026). Pearson correlation analysis was performed to calculate correlations between all genes and metabolites after z-score normalization, with significant correlations screened by |CC| > 0.80 and *p* < 0.05. Overlapped pathways between transcriptomic and metabolomic datasets were identified and visualized by a Venn diagram. KEGG enrichment was assessed by Fisher’s exact test with Bonferroni correction (*P*_bonferroni ≤ 0.05). Using KEGG Markup Language, the igraph R package (v3.6.1) was used to construct and visualize pathway-gene-metabolite interaction networks, from which the most significantly enriched pathways were selected for presentation.

### 2.6. Data Analysis

Comparisons of feeding puncture counts and oviposition amounts of *L. trifolii* on bitter gourd versus kidney bean, together with the effects of the leaf extract on growth and developmental parameters, were analyzed using independent-samples *t*-tests with SPSS 16.0 (SPSS, Chicago, IL, USA), and statistical significance was set at *p* < 0.05. Correlation analyses between qPCR and RNA-seq data, as well as the graphical presentation of experimental results, were performed using GraphPad Prism (version 8.0.1). Relative expression levels of target genes were calculated using the 2^−ΔΔCt^ method [30].

## 3. Results

### 3.1. Feeding and Oviposition of L. trifolii on Different Host Plants

**Figure 1:**
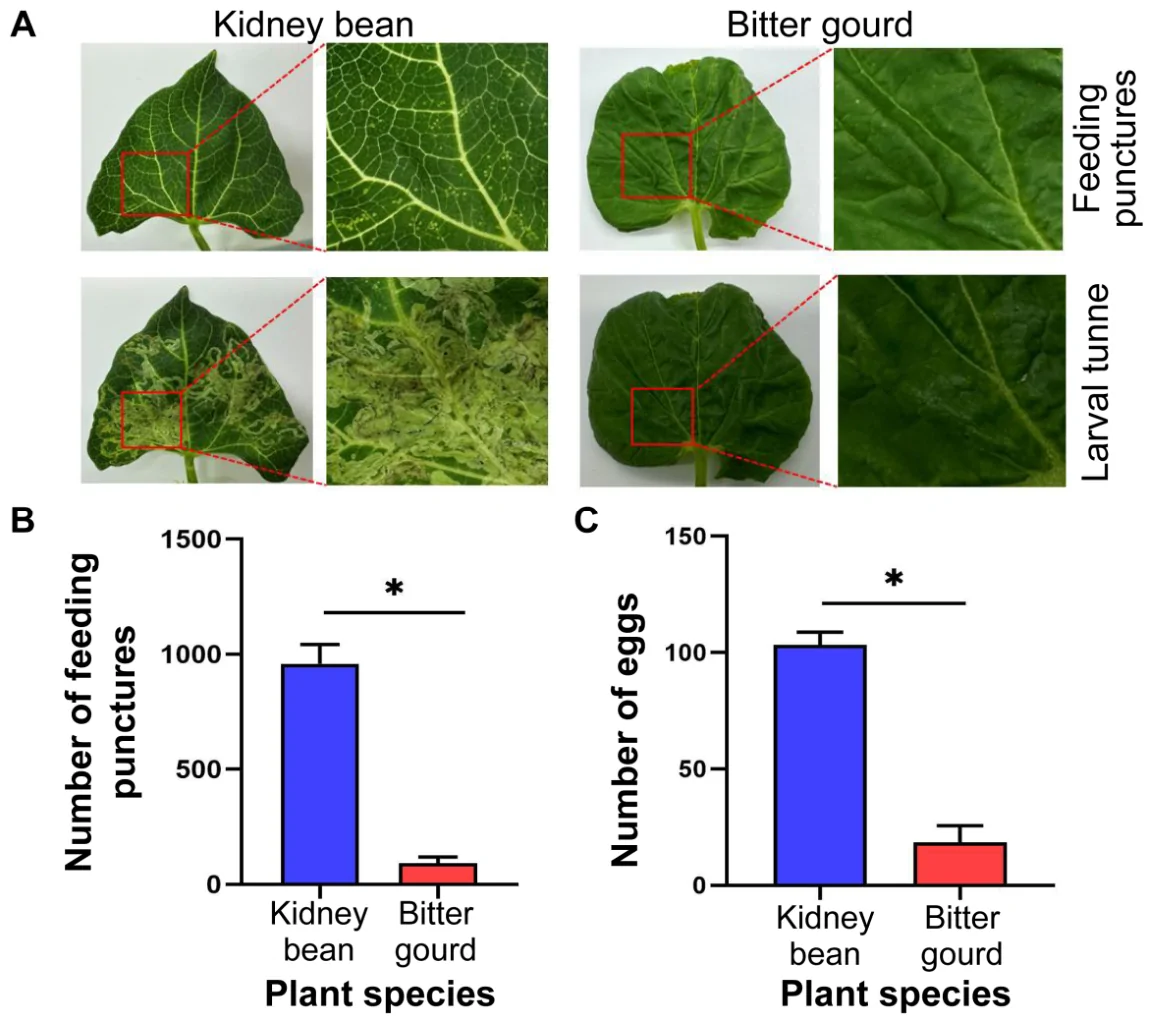
Feeding and oviposition of *Liriomyza trifolii* on kidney bean and bitter gourd. (**A**) Representative leaves showing feeding punctures and larval mines; (**B**) number of feeding punctures; (**C**) number of eggs laid. Asterisks indicate significant differences between kidney bean and bitter gourd (* *p* < 0.05).

The two host plants exerted contrasting effects on the development of *L. trifolii*. On bitter gourd, the insect failed to complete a full generation, with no leaf mines observed on bitter gourd, although minor feeding and oviposition were observed (Figure 1A). Consistently, both the number of feeding punctures (Figure 1B) and eggs laid (Figure 1C) on kidney bean were significantly higher than those on bitter gourd (feeding punctures: *t* = 10.02, *p* < 0.001; eggs: *t* = 9.34, *p* < 0.001).

### 3.2. Toxic Effects of Bitter Gourd Leaf Extract Against L. trifolii

The insecticidal activity of bitter gourd extract against *L. trifolii* is presented in Table 1. The insecticidal activity of the bitter gourd extract against *L. trifolii* increased with increasing concentrations. The regression equation for the relationship between extract concentration and insecticidal activity at 24 h was y = 1.022x − 0.718, with a correlation coefficient R^2^ = 0.831. The calculated median lethal concentration (LC_50_) was 5.042 mg/mL.

### 3.3. Inhibitory Effects of Bitter Gourd Extract on the Growth and Development of L. trifolii

**Figure 2:**
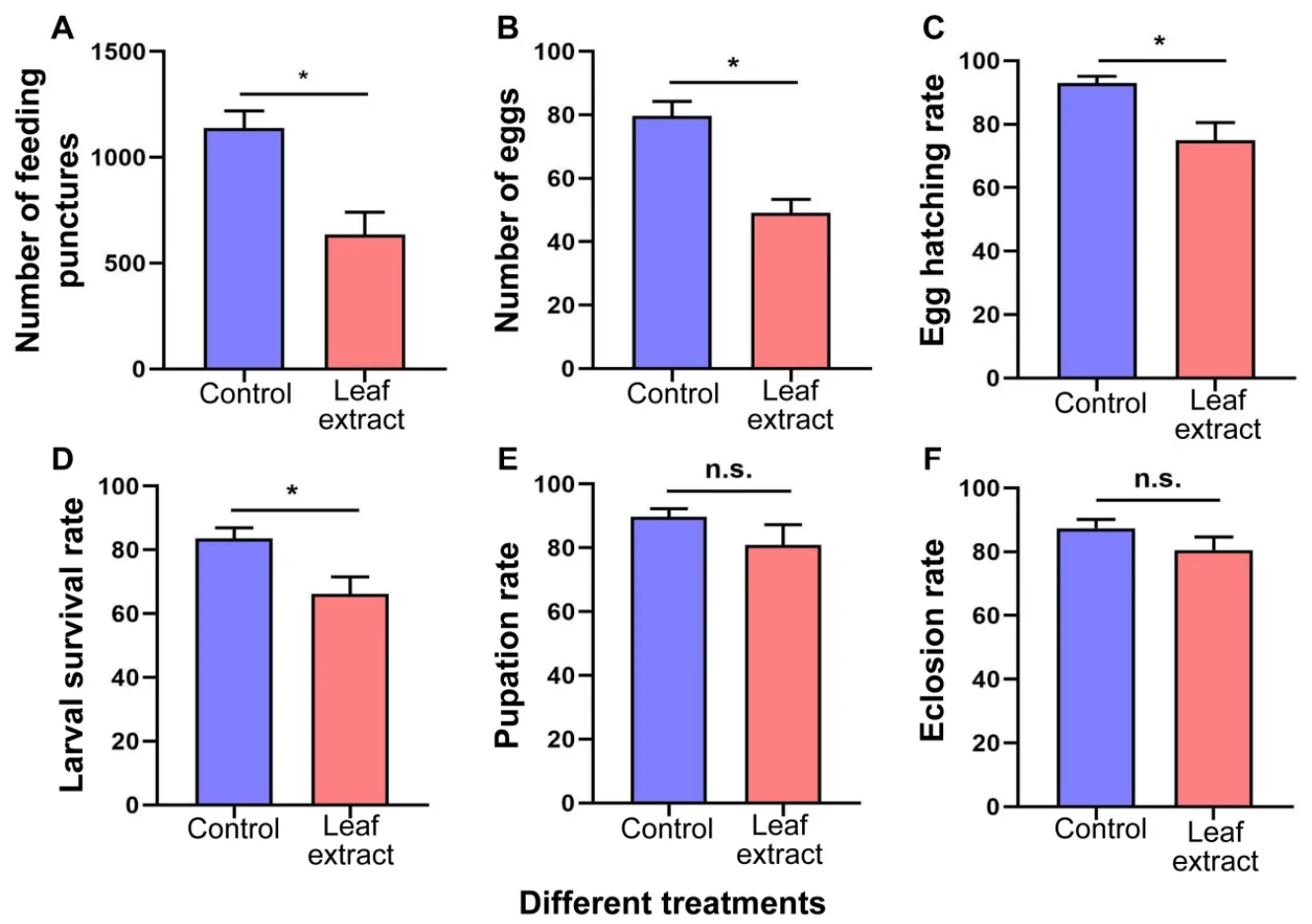
Effects of bitter gourd leaf extract on the growth and development of *Liriomyza trifolii*. (**A**) Number of feeding punctures; (**B**) number of eggs laid; (**C**) egg hatching rate; (**D**) larval survival rate; (**E**) pupation rate; (**F**) emergence rate. Asterisks indicate significant differences between the control and extract treatment groups (* *p* < 0.05); n.s., not significant.

In this experiment, the bitter gourd extract served as the treatment group, while a solution without the extract was used as the control, to investigate the effects of the extract on the number of feeding punctures, oviposition, and developmental parameters at various life stages of *L. trifolii*. The results showed that the number of feeding punctures on kidney bean seedlings in the control group was significantly higher than that in the bitter gourd extract treatment group (*t* = 3.77; *p* = 0.0037), indicating that the extract treatment significantly reduced the feeding behavior of *L. trifolii* on bean seedlings (Figure 2A). Oviposition in the control group was significantly higher than that in the treatment group (*t* = 4.82; *p* = 0.0007), demonstrating that the LC_50_ concentration of bitter gourd extract exerted a marked inhibitory effect on the oviposition capacity of *L. trifolii*, significantly reducing egg production and thereby lowering the population base of the next generation (Figure 2B). The egg hatching rate in the treatment group was significantly lower than that in the control group (*t* = 3.01; *p* = 0.0132), indicating that the extract interfered with normal egg hatching, reduced egg viability, and exerted significant inhibitory effects during the early developmental stage (Figure 2C). The larval survival rate in the treatment group was significantly lower than that in the control group (*t* = 2.77; *p* = 0.0198), suggesting that the extract had a notable negative impact on larval survival, effectively reducing survival during the larval stage and hindering subsequent population development (Figure 2D). No statistically significant difference was observed in pupation rate between the treatment and control groups (*t* = 1.28; *p* = 0.2310), with both groups showing similar pupation proportions, indicating that the extract did not markedly interfere with the larval-to-pupal transition (Figure 2E). Similarly, no significant difference was found in emergence rate between the two groups (*t* = 1.38; *p* = 0.1964), with comparable adult emergence proportions, suggesting that the LC_50_ concentration of the extract did not substantially affect the emergence process of pupae, and individuals that survived to the pupal stage could still emerge normally (Figure 2F).

### 3.4. Transcriptomic Analysis and Validation of L. trifolii Treated with Bitter Gourd Extract

**Figure 3:**
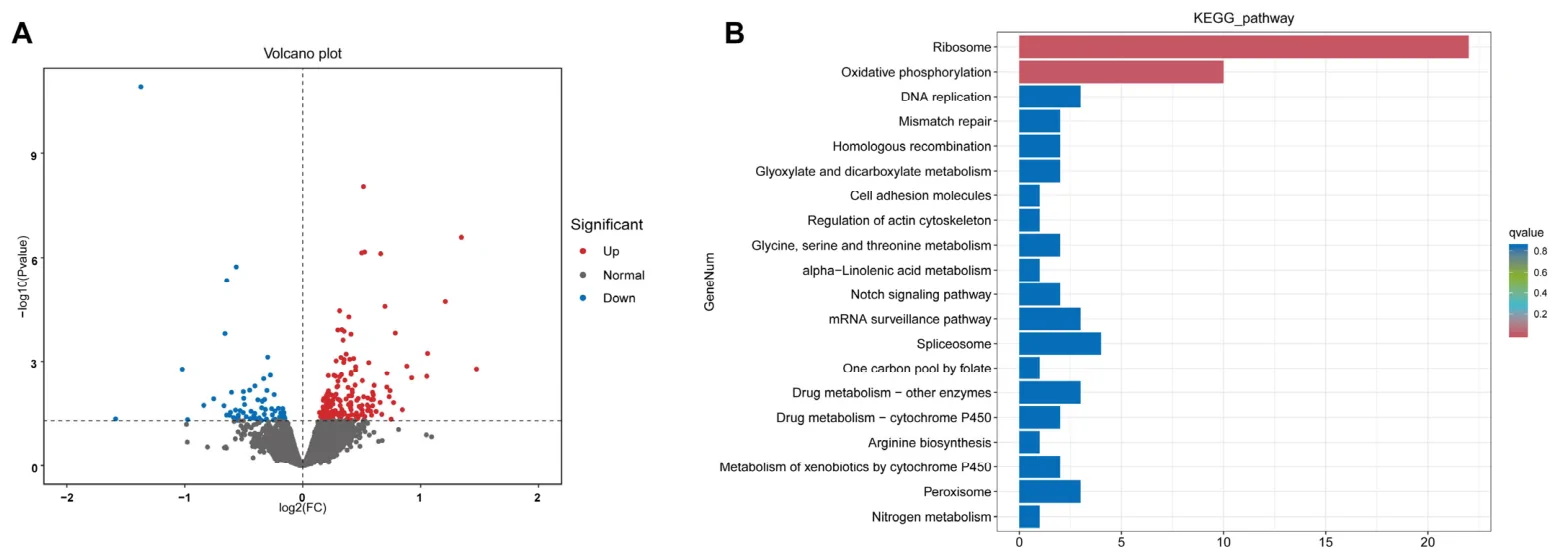
Transcriptomic responses of *Liriomyza trifolii* to bitter gourd leaf extract treatment. (**A**) Volcano plot of differentially expressed genes (DEGs); (**B**) KEGG pathway enrichment analysis of DEGs.

In the transcriptomic analysis, pairwise comparison between the CK group and the LC_50_ treatment group identified a total of 254 differentially expressed genes (DEGs), of which 187 were upregulated and 67 were downregulated (Table S2; Figure 3A). To elucidate the functional roles of the DEGs under LC_50_ treatment, the identified DEGs were annotated against the KEGG, COG, and GO databases to uncover key biological processes related to stress response, metabolic regulation, and detoxification.

KEGG enrichment analysis (Figure 3B) showed that the “Ribosome” pathway exhibited the highest enrichment and significance, with overall significant upregulation of the related genes. The “Oxidative phosphorylation” pathway was the second most enriched, also showing a significant upregulation trend. The coordinated activation of these two pathways suggests that protein synthesis and energy metabolism are highly active in the samples, indicating a state of elevated metabolism and proliferation. Other pathways were mainly concentrated in supportive functions such as DNA replication and repair (“DNA replication”, “Mismatch repair” and “Homologous recombination”), RNA splicing and processing (“Spliceosome”), and detoxification metabolism (“Drug metabolism—other enzymes”, “Drug metabolism—cytochrome P450”, “Metabolism of xenobiotics by cytochrome P450”), as well as in signal transduction (“Notch signaling pathway”, “Cell adhesion molecules”, “Regulation of actin cytoskeleton”) and amino acid/small-molecule metabolism.

COG annotation results revealed that among all 26 functional categories, the top three most enriched were: “Translation, ribosomal structure and biogenesis,” “Posttranslational modification, protein turnover, chaperones,” and “Cell wall/membrane/envelope biogenesis” (Figure S4). In the GO classification, each annotated DEG was assigned to three categories: biological process, cellular component, and molecular function. Within these categories, “cellular process,” “binding,” and “cellular anatomical entity” were the predominant terms, respectively (Figure S4). The top 30 differentially expressed genes (15 up-regulated and 15 down-regulated) screened from all DEGs under LC_50_ treatment are presented in Table 2. Among the up-regulated genes, functional annotations revealed enrichment in protein synthesis, stress responses, nitrogen metabolism, and detoxification, with significant up-regulation of ribosomal protein genes and transposon-associated elements, alongside increased expression of Vitellin-degrading protease, Sentrin-specific protease, and Multifunctional methyltransferase subunit TRM112-like protein, which collectively suggest activation of protein turnover and RNA modification. Notably, the up-regulation of Glutamine synthetase 1 and UDP-glycosyltransferase UGT5 implies a metabolic shift toward enhanced amino acid assimilation and xenobiotic clearance. In contrast, the down-regulated genes were primarily associated with transcriptional regulation, fatty acid oxidation, immune defense, and nucleic acid metabolism, as evidenced by the reduced expression of Helix-loop-helix protein 13, Histone deacetylase HDAC1, Protein spaetzle 4, Probable peroxisomal acyl-coenzyme A oxidase 1, and Single-stranded DNA-binding protein. These coordinated alterations, activation of protein synthesis and detoxification coupled with suppression of immune and lipid metabolic pathways, indicate that *L. trifolii* mounts a compensatory stress response while suffering from impaired adaptability, collectively revealing a complex molecular regulatory network underlying the insect’s response to bitter gourd extract stress (Table 2).

To validate the reliability of the transcriptomic data, eight differentially expressed genes were selected and subjected to qRT-PCR validation. Overall, the expression trends of the differentially expressed genes were consistent with the transcriptomic results. A correlation analysis between the qPCR data and RNA-seq data was performed. To ensure a normalized comparison, the data compared were the fold changes between the LC_50_ treatment group and the control group. The results show a significant correlation between the two datasets, with an R^2^ of 0.59 and a *p*-value of 0.03. These findings indicate that the reliability and accuracy of the transcriptomic sequencing data have been confirmed (Figure S5).

### 3.5. Metabolomic Analysis of L. trifolii Treated with Bitter Gourd Extract

**Figure 4:**
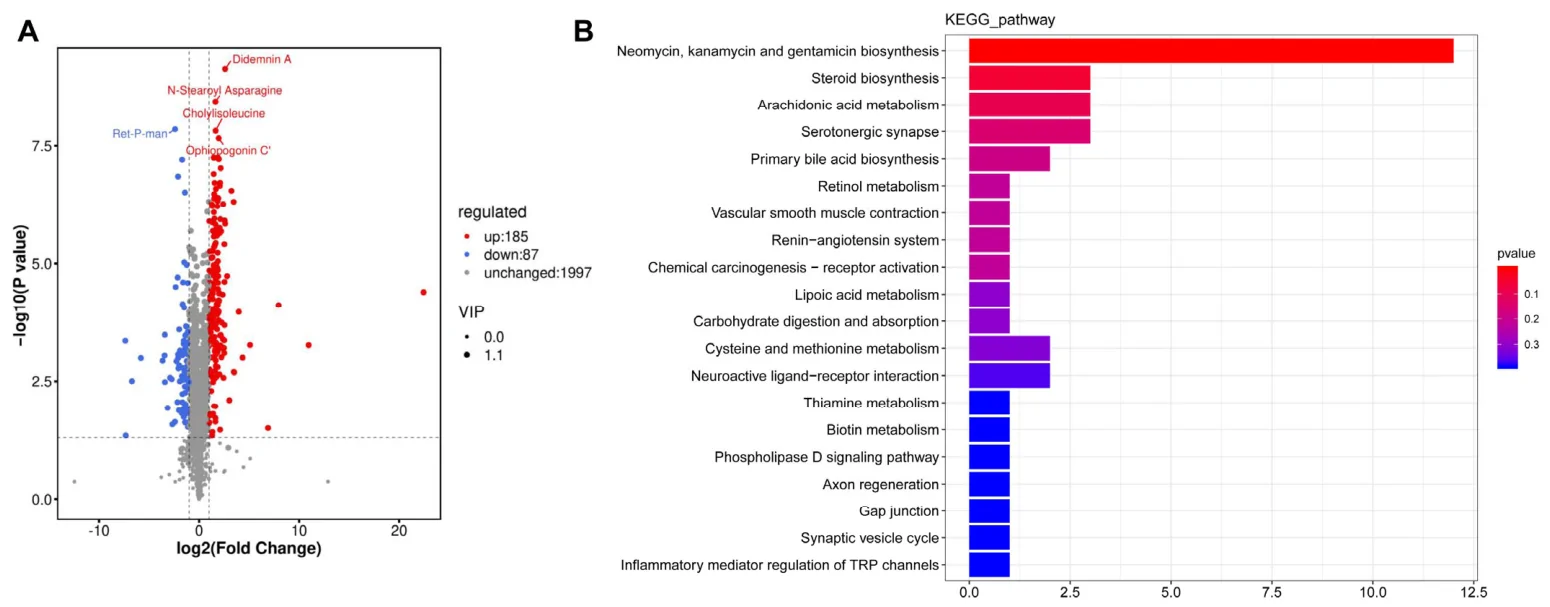
Metabolomic profiling of *Liriomyza trifolii* in response to bitter gourd extract. (**A**) Volcano plot of differential metabolites; (**B**) KEGG pathway enrichment analysis of differential metabolites.

After the annotation and classification of differentially expressed metabolites, the results showed that in LC-MS untargeted metabolomics, a total of 272 significantly differential metabolites were detected (185 upregulated and 87 downregulated), along with 1997 non-significant metabolites (Table S3). The top five identified differential metabolites with the lowest *p*-values are labeled in the plot, allowing for intuitive identification of core differential metabolites. Representative upregulated metabolites included deoxyinosine, hypoxanthine, and orotidine, most of which are involved in purine metabolism pathways. Representative downregulated metabolites included clavaminic acid, S-adenosylmethioninamine, and indole-3-acetyl glutamic acid, which are associated with cysteine and methionine metabolism, and arginine and proline metabolism pathways (Figure 4A).

Based on the KEGG database, pathway enrichment annotation and classification were performed for the differential metabolites between the LC_50_ treatment group and the control group. The results revealed that the most significantly enriched pathway was neomycin, kanamycin and gentamicin biosynthesis, followed by steroid biosynthesis, arachidonic acid metabolism, primary bile acid biosynthesis, retinol metabolism, carbohydrate digestion and absorption, serotonergic synapse, and neuroactive ligand-receptor interaction, all of which were significantly upregulated. Meanwhile, vascular smooth muscle contraction and lipoic acid metabolism pathways showed notable downregulation. In addition, thiamine metabolism and cysteine and methionine metabolism also exhibited a certain degree of enrichment, indicating that steroid synthesis, lipid metabolism, amino acid metabolism, and neurosignaling-related pathways play important roles in the metabolic responses of samples to LC_50_ treatment, representing the core metabolic regulatory pathways under this treatment condition (Figure 4B).

The top 30 significantly differential metabolites (15 up-regulated and 15 down-regulated) screened from all DEMs under LC_50_ treatment are presented in Table 3. Among the up-regulated metabolites, terpenoid lipids were the most prominent category, including 8′-apo-β-caroten-8′-ol, 4,4-dimethyl-5α-cholesta-8-en-3β-ol, 28-glucosyl-19(29)-dehydroursolic acid 3-arabinoside, maslinic acid 3-O-β-D-glucoside, and deoxyloganic acid, whose significant accumulation may reflect detoxification or sequestration of exogenous terpenoids; the concurrent up-regulation of 22alpha-hydroxy-campest-4-en-3-one, 2′,3′-dideoxyuridine, glycyl-serine, and didemnin A further suggested disturbances in hormone and nucleic acid metabolism alongside activated protein turnover. In contrast, the down-regulated metabolites were predominantly glycerolipids, fatty acyls, purine nucleosides, and terpenoid lipids, as evidenced by reduced levels of DG(14:1(9Z)/18:0/0:0), (-)-jasmonic acid, PGD2 ethanolamide, 1-methylguanosine, Ret-P-man, and comuside, indicating suppression of lipid storage, fatty acid synthesis, stress signaling, immune regulation, and purine metabolism. Collectively, these coordinated metabolic alterations, activation of terpenoid detoxification and amino acid metabolism coupled with inhibition of lipid and nucleotide pathways, reveal that *L. trifolii* mounts a compensatory detoxification response while suffering from impaired energy metabolism and immune defense, collectively delineating a comprehensive metabolic network underlying the insect’s response to bitter gourd extract stress (Table 3).

### 3.6. Correlation Analysis Between Differentially Expressed Genes and Differentially Expressed Metabolites

**Figure 5:**
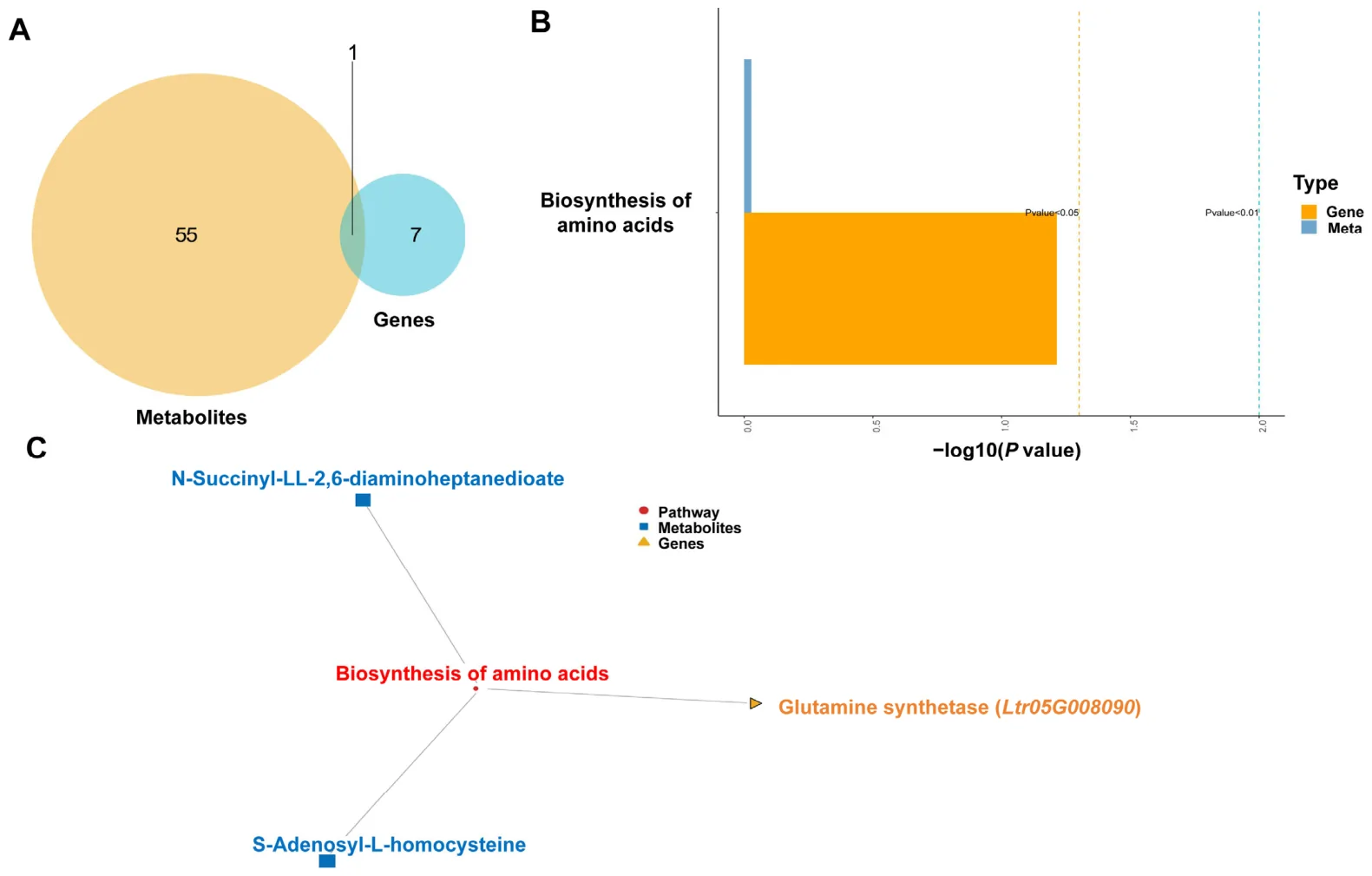
Integrated multi-omics analysis of *Liriomyza trifolii* in response to bitter gourd extract. (**A**) Venn diagram of KEGG pathways shared between DEGs and DEMs. The diagram indicates that 55 pathways were uniquely associated with metabolites, 7 pathways were uniquely associated with genes, and 1 pathway was shared between the two datasets; (**B**) KEGG enrichment bar chart of DEGs and DEMs; (**C**) correlation network of key DEGs and DEMs in the “Biosynthesis of amino acids” pathway.

To further explore the biological functions and metabolic pathways of DEGs and DEMs in *L. trifolii* under bitter gourd extract treatment, KEGG pathway enrichment analysis was performed on both datasets. By comparing the pathways involved in the transcriptomic genes and the pathways involved in the metabolomic metabolites, the number of co-participated pathways was obtained (Table S4). The Venn diagram and KEGG enrichment plot showed that only one pathway, “Biosynthesis of amino acids,” was commonly identified (Figure 5A,B).

Further analysis of the KGML files from the KEGG pathways provided detailed pathway component information, enabling the construction of a network relationship among differential genes, differential metabolites, and their associated pathways. In this study, under bitter gourd extract treatment, two differential metabolites-S-adenosyl-L-homocysteine and *N*-Succinyl-LL-2,6-diaminoheptanedioate-and one differentially expressed gene, glutamine synthetase (*Ltr05G008090*), were identified as having significant correlation relationships. S-Adenosyl-L-homocysteine (log_2_FC: 1.36; *p* value: <0.001; VIP: 1.19), commonly referred to as SAH or AdoHcy, is a core molecule that acts as a “metabolic regulator” in organisms, playing a vital role particularly in the growth, development, and senescence of insects. *N*-Succinyl-LL-2,6-diaminoheptanedioate (log_2_FC: −1.60; *p* value: 0.0016; VIP: 1.19) is a key metabolic intermediate in the bacterial lysine biosynthesis pathway; however, insects completely lack the diaminopimelate pathway and must obtain lysine from their diet, as lysine is an essential amino acid for insects. Notably, the differential gene glutamine synthetase (*Ltr05G008090*) functions as an efficient “nitrogen processing center” in insects, converting toxic waste into useful raw materials and profoundly influencing insect growth, reproduction, and stress adaptation (Figure 5C).

## 4. Discussion

Plant-derived bioactive substances, which interfere with pest feeding, development, and reproduction through the behavioral and toxic effects of secondary metabolites, are increasingly important in green pest management; plant extracts, as key sources of botanical insecticides, offer environmental compatibility, low residue, low resistance risk, and synergism with other agents, advantages that address many limitations of conventional pesticides and support sustainable pest control [10,12,14]. Thus, developing novel green insecticides from plant extracts is a research priority for balancing effective control with human and environmental safety. Bitter gourd (*Momordica charantia*) stands out as an edible, medicinal, and insecticidal plant, with confirmed resistance against multiple pests, including *Liriomyza* leafminers [17,20,31,32]. Despite its considerable potential, existing studies remain largely phenotypic, with limited exploration of the underlying molecular mechanisms.

Host plant secondary metabolites critically mediate herbivorous insect host selection, development, and population dynamics [33]. In our host suitability assays, *L. trifolii* showed strong oviposition adaptability for kidney bean over bitter gourd, with significantly more eggs and feeding punctures on bean seedlings, and failed to complete a full generation on bitter gourd, confirming its natural oviposition repellency and non-host resistance. This preference likely reflects the rich terpenoids and phenolics in bitter gourd that influence insect olfactory and feeding behavior [22,23,34,35]. Notably, our findings align with previous reports on *L. sativae*, which also exhibits low feeding and oviposition adaptability for bitter gourd and rarely forms mines on its leaves [21]. This cross-species consistency points to a general resistance of bitter gourd against *Liriomyza* spp., likely underpinned by conserved secondary metabolites, with cucurbitacin B identified as a key bioactive component [21]. Toxicity bioassays of the ethanol extract of bitter gourd revealed significant dose-dependent insecticidal activity. At the LC_50_ concentration, the extract markedly reduced the number of feeding punctures, oviposition amount, egg hatching rate, and larval survival rate, while pupation and emergence rates remained unaffected, indicating a stage-specific inhibitory pattern. Notably, the LC_50_ concentration applied in the present study was obtained through the cotton-wick feeding method and served exclusively as an experimental treatment concentration in the developmental assay, not as an LC_50_ value derived from the leaf-dipping method. This experimental design was intentionally adopted to evaluate the persistent effects of the extract on adult feeding behavior, oviposition, and later life stages, for which the leaf-dipping method is best suited, and is also aligned with the long-term goal of advancing field applications of bitter gourd extract. This differential susceptibility likely reflects the extract’s primary action on adult behavior and early larval development, whereas pupae, with altered cuticular structure and metabolic traits, show greater tolerance. Such stage dependence aligns with known modes of action of bitter gourd metabolites: momordicin I disrupts midgut epithelial cells and inhibits digestive enzymes [36], and sesquiterpenoids boost oxidative stress by interfering with superoxide dismutase [37], effects most potent in actively feeding larvae but attenuated in metabolically quiescent pupae. From a management standpoint, the strong oviposition suppression enables early-stage “source control” by reducing the next-generation population base, while larval mortality directly curtails field damage potential. The lack of effect on pupation and emergence, however, suggests the extract is not a standalone late-stage tool; instead, it pairs well with complementary tactics targeting pupal or adult stages.

To elucidate the molecular mechanisms underlying the insecticidal activity of bitter gourd extract against *L. trifolii*, we performed integrated transcriptomic and metabolomic analyses on adults that survived 24 h post-treatment. Given that these surviving individuals may represent a relatively tolerant subpopulation, the observed molecular alterations are more likely to reflect compensatory responses, detoxification processes, or tolerance mechanisms rather than the direct causes of mortality. Nevertheless, our findings provide important molecular evidence for understanding the interaction mechanisms between bitter gourd and *Liriomyza* species, as well as the principles underlying plant-based pest management. KEGG enrichment analysis of differentially expressed genes revealed that the “Ribosome” and “Oxidative phosphorylation” pathways were predominantly upregulated, whereas the “Drug metabolism” pathways were mainly downregulated. Among the upregulated genes, ribosomal protein L39 (RPL39) and cytochrome c oxidase subunit 7A1 (COX7A1) supported the activation of protein synthesis and energy metabolism. RPL39 has been shown to play a role in the development of insecticide resistance in *Culex pipiens pallens* [38], while the upregulation of COX7A1 leads to the activation of the “Oxidative phosphorylation” pathway. This may be associated with the surge in energy demand when insects respond to the toxic stress of bitter gourd extract. Glutamine synthetase and UDP-glycosyltransferase UGT5 were upregulated, suggesting enhanced nitrogen assimilation and xenobiotic detoxification. The upregulation of glutamine synthetase is consistent with the expression pattern of this enzyme in response to xenobiotic stress in the Asiatic honeybee (*Apis cerana cerana*) [39]. Similarly, the upregulation of UGT5 aligns with the activation of UGTs in pyrethroid-resistant populations of *Spodoptera litura* and in the detoxification of gossypol in *Helicoverpa armigera* [40,41], suggesting that these genes jointly participate in the coordinated response of nitrogen assimilation and detoxification metabolism when insects cope with chemical stress. The activation of transposon-related elements and Sentrin-specific protease reflected genomic stress responses and accelerated protein turnover; the activation of transposon-related elements is consistent with findings in *Plutella xylostella*, where transposons have been reported to participate in stress responses and adaptive evolution [42]. In contrast, the downregulation of histone deacetylase HDAC1 is consistent with the phenotypes of transcriptional repression and developmental defects observed following HDAC1 knockdown in *Aedes aegypti* [43]; the downregulation of Spätzle 4 suggests suppression of the Toll immune pathway, which aligns with findings in *Mythimna separata* where Spätzle 4 regulates immune responses [44]; and the downregulation of peroxisomal acyl-coenzyme A oxidase 1 directly leads to reduced fatty acid β-oxidation. Although cytochrome P450 was the sole detoxification gene significantly upregulated, its singular expression was insufficient to counteract the toxic stress induced by bitter gourd extract, consistent with previous reports on the contribution of the P450 gene cluster to phytochemical detoxification in *Helicoverpa armigera* [45]. When the toxic load exceeds the insect’s limited detoxification capacity, it ultimately results in toxic accumulation and physiological impairment. GO enrichment terms, dominated by “Cellular process”, “Binding”, and “Cellular anatomical entity”, reflected multi-dimensional cellular disruption.

Metabolomic analysis detected 272 significantly differential metabolites. Among the upregulated metabolites, terpenoid lipids were the most prominent (e.g., 8′-apo-β-caroten-8′-ol, maslinic acid 3-O-β-D-glucoside, deoxyloganic acid), whose accumulation may reflect detoxification or sequestration of exogenous terpenoids, a phenomenon similar to the detoxification strategy employed by *Chrysomela tremulae*, which utilizes tryptophan metabolites to conjugate and excrete salicinoids from poplar trees [46]. The upregulation of purine metabolites such as hypoxanthine and deoxyinosine indicated enhanced energy synthesis and repair [47], consistent with oxidative phosphorylation activation, which aligns with the central role of purine metabolism in energy synthesis under stress in overwintering *Apis cerana cerana* [48]. The marked upregulation of 22alpha-Hydroxy-campest-4-en-3-one suggested hormonal disruption, consistent with reports that phytochemicals affect insect development by directly acting on the ecdysone receptor [49] or interfering with cholesterol metabolism (saponin-related studies); however, its excessively high fold change suggests an imputed/floor value rather than a genuine measurement, and further studies are needed to verify this. The accumulation of 2′,3′-dideoxyuridine and glycyl-serine reflected pyrimidine metabolic disturbance and enhanced protein degradation. Among the downregulated metabolites, decreased diglycerides indicated suppressed lipid storage; reduced levels of (-)-jasmonic acid and PGD2 ethanolamide weakened stress signaling and immune regulation, the central role of eicosanoid signaling molecules in insect immunity has been well established [50]; downregulation of 1-methylguanosine suggested inhibited purine metabolism and nucleic acid synthesis; and decreased amino acid derivatives (e.g., indole-3-acetyl glutamic acid, S-adenosylmethioninamine) pointed to impaired amino acid metabolism, directly linking to reduced protein synthesis and the observed declines in egg hatching and larval survival rates [51]. KEGG enrichment of steroid biosynthesis and arachidonic acid metabolism pathways further confirmed multi-pathway perturbation, similar to the multi-metabolic pathway reprogramming pattern observed in *Propylea japonica* under heat stress [52].

Integrated analysis identified “Biosynthesis of amino acids” as the sole common KEGG pathway between the two datasets, with the glutamine synthetase (GS) gene showing significant correlations with S-adenosyl-L-homocysteine (SAH) and *N*-Succinyl-LL-2,6-diaminoheptanedioate, although these two metabolites were not among the top-15 lists of up-regulated or down-regulated metabolites. Glutamine synthetase (GS) is a pivotal enzyme catalyzing the synthesis of glutamine (Gln). As an essential precursor for amino acid biosynthesis, glutamine participates in nitrogen metabolism and is critical for insect growth, development and reproduction. As a core enzyme responsible for ammonia detoxification and nitrogen assimilation in insects, GS provides substrates for amino acid and protein synthesis. The expression level of GS directly affects nitrogen metabolism and protein biosynthesis [53]. In *Bombyx mori*, *BmGS1* participates in fertilization [54]. In *Rhopalosiphum padi*, *RpGS* regulates aphid fecundity by affecting symbionts [55]. In *Bactrocera dorsalis*, BdGS mediates larval development and pupation by modulating ecdysteroid biosynthesis, and this gene also influences female fecundity [56,57]. These studies demonstrate that GS acts as a multifunctional regulator in insects and serves as a promising target for pest control.

Although direct studies on *N*-succinyl-LL-2,6-diaminoheptanedioate in insects remain scarce in the current literature, the lysine biosynthetic pathway in which it is involved has been demonstrated to play a critical role in the cooperative metabolism between insects and their symbiotic microorganisms, and this pathway is indispensable for host development [58]. The metabolite and pathway changes observed in our study highlight the marked effects of the bitter gourd leaf extract. However, whether this metabolite is functionally linked to symbiont coordination in *L. trifolii* warrants further investigation. S-adenosyl-L-homocysteine, as a product of trans-methylation reactions, regulates methylation processes through feedback inhibition of methyltransferase activity. In *Sarcophaga peregrina*, SAH accumulation selectively inhibits the activation of defense protein genes but does not affect HSP70 expression [59]; in *Aedes aegypti*, SAH feedback inhibits juvenile hormone acid methyltransferase (JHAMT) activity, affecting hormone synthesis and developmental regulation [60]. These metabolic disturbances, combined with insufficient detoxification, ultimately result in developmental failure. These findings reveal, at the molecular level, that bitter gourd extract exerts systemic toxicological effects through the disruption of multiple metabolic pathways, including amino acid biosynthesis, nitrogen metabolism, methylation regulation, and hormone synthesis in insects. These multi-omics findings are underpinned by a solid chemical basis. Bitter gourd secondary metabolites, including alkaloids (e.g., momordicin I, charantin), terpenoids (cucurbitane-type triterpenoid saponins, sesquiterpenoids), and flavonoids, act through toxicity, growth inhibition, and behavioral interference. Momordicin I disrupts midgut cells and inhibits digestive enzyme activity [36,61]; sesquiterpenoids enhance oxidative stress [37]; and flavonoids synergize with other components [62]. The insect resistance of bitter gourd relies not only on volatile repellents but also on a comprehensive chemical defense system formed by its abundant secondary metabolites [63]. Alkaloids are key insecticidal components; momordicin-type compounds exhibit significant antifeedant and toxic effects against chewing pests [64], and their modes of action are consistent with the reduced feeding punctures and decreased egg hatching rates observed in this study. Among terpenoids, cucurbitane-type triterpenoid saponins inhibit α-amylase and β-glucosidase, interfering with carbohydrate metabolism, while sesquiterpenoids exert growth inhibitory effects on pests and enhance oxidative stress [37], consistent with the oxidative phosphorylation activation and peroxisome pathway down-regulation observed in our transcriptome. Flavonoids possess antioxidant and antimicrobial activities and synergize with alkaloids and terpenoids to improve overall efficacy [62]; for example, quercetin and kaempferol inhibit aphid feeding, prolong development, and reduce oviposition [65]. The multi-mechanistic and synergistic actions of bitter gourd secondary metabolites, through toxicity, antifeedant effects, growth inhibition, and enzyme activity interference, comprehensively explain the complex stress responses observed: *L. trifolii* faces not a single compound, but a multi-component, synergistically acting chemical defense system.

## 5. Conclusions

In conclusion, although *L. trifolii* is a polyphagous pest, it exhibits significant oviposition inhibition on bitter gourd, and the ethanol extract of bitter gourd shows significant dose-dependent insecticidal activity and stage-specific growth and development inhibitory effects. The control effects of bitter gourd extract against *L. trifolii* are achieved primarily through interference with material and energy metabolism, suppression of detoxification system function, and disruption of early developmental processes, with the “Biosynthesis of amino acids” pathway and key molecules such as the glutamine synthetase gene *Ltr05G008090* serving as important candidate targets. The findings of this study may offer useful molecular insights into the interaction between bitter gourd and *Liriomyza* species, and may contribute to a better understanding of plant-based pest management strategies.

## Figures and Tables

**Table 1:** The toxicity sensitivity of *Liriomyza trifolii* adults to bitter gourd leaf extract.

**Bitter Gourd Leaf Extract**	**LC_50_** **(mg/mL)**	**95% Confidence Interval**	**Linear Fit Regression Equation**	**R^2^**	**Chi-** **Square**	***p*** **Value**
	5.042	3.866~6.580	y = 1.022x − 0.718	0.831	12.760	0.957

**Table 2:** Top 15 up-regulated and down-regulated differentially expressed genes (DEGs) in *Liriomyza trifolii* under bitter gourd extract treatment.

#ID	*p* Value	log_2_ FC	Swissprot Annotation	NR_Annotation
Up-regulated				
*NewGene_1100*	0.002	1.47	--	hypothetical protein EVAR_64979_1
*Ltr05G005380*	<0.001	1.21	Retrovirus-related Pol polyprotein from transposon 297	ubiquitin carboxyl-terminal hydrolase puf-like
*NewGene_965*	<0.001	1.06	Copia protein	Copia protein
*Ltr04G004500*	0.003	1.05	--	basement membrane-specific heparan sulfate proteoglycan core protein isoform X8
*NewGene_192*	0.003	0.92	Sentrin-specific protease	PREDICTED: uncharacterized protein LOC108364159
*Ltr03G021330*	0.001	0.88	--	uncharacterized protein LOC105224552
*LtrUnG013390*	0.025	0.84	Vitellin-degrading protease	CLIP domain-containing serine protease 14D isoform X2
*Ltr05G017450*	<0.001	0.78	--	cytochrome c oxidase subunit 7A1, mitochondrial
*Ltr01G004150*	0.046	0.75	--	PREDICTED: uncharacterized protein LOC108358328 isoform X1
*Ltr01G012840*	0.007	0.74	Multifunctional methyltransferase subunit TRM112-like protein	multifunctional methyltransferase subunit TRM112-like protein
*Ltr02G003400*	0.011	0.73	--	uncharacterized protein LOC105209288
*Ltr05G008090*	0.006	0.71	Glutamine synthetase 1, mitochondrial	PREDICTED: glutamine synthetase 1, mitochondrial-like
*Ltr05G009230*	0.033	0.67	UDP-glycosyltransferase UGT5	UDP-glucuronosyltransferase 2A3
*Ltr03G015170*	<0.001	0.66	60S ribosomal protein L39	sxc
*Ltr03G015550*	0.015	0.66	--	uncharacterized protein LOC119679229 isoform X1
Down-regulated				
*Ltr01G004330*	<0.001	−1.37	Retrovirus-related Pol polyprotein from transposon opus	uncharacterized protein LOC121404470
*NewGene_964*	0.002	−1.02	Copia protein	Copia protein
*Ltr01G009260*	0.047	−0.98	--	uncharacterized protein LOC123257649
*Ltr01G006410*	0.018	−0.84	Retrovirus-related Pol polyprotein from transposon 412	unnamed protein product
*Ltr02G008310*	0.012	−0.75	Helix-loop-helix protein 13	basic helix-loop-helix transcription factor amos
*NewGene_46*	0.019	−0.67	--	uncharacterized protein LOC112692539
*Ltr05G009530*	<0.001	−0.66	Retrovirus-related Pol polyprotein from transposon 297	ubiquitin carboxyl-terminal hydrolase puf-like
*NewGene_736*	0.035	−0.65	--	env-like protein
*Ltr01G000530*	<0.001	−0.64	--	mucin-2-like
*Ltr01G001030*	0.029	−0.61	Histone deacetylase HDAC1	histone deacetylase HDAC1
*Ltr05G005520*	0.008	−0.60	Protein spaetzle 4	protein spaetzle 4
*Ltr05G006780*	0.050	−0.57	Probable peroxisomal acyl-coenzyme A oxidase 1	probable peroxisomal acyl-coenzyme A oxidase 1
*Ltr02G023740*	<0.001	−0.56	Single-stranded DNA-binding protein, mitochondrial	single-stranded DNA-binding protein, mitochondrial
*Ltr02G012150*	0.049	−0.55	Endoribonuclease CG2145	poly(U)-specific endoribonuclease homolog
*Ltr05G006260*	0.035	−0.55	Basic helix-loop-helix transcription factor amos	heart- and neural crest derivatives-expressed protein 2

**Table 3:** Top 15 up-regulated and down-regulated differential metabolites identified in *Liriomyza trifolii* under bitter gourd extract treatment.

#ID	Name	log_2_ FC	*p* Value	VIP	Taxonomy
Up-regulated					
pos_3165	22alpha-Hydroxy-campest-4-en-3-one	22.44	<0.001	1.19	Steroids and steroid derivatives
neg_3334	PA(18:0/TXB2)	10.95	<0.001	1.19	Glycerophospholipids
pos_3161	8′-Apo-b-caroten-8′-ol	7.95	<0.001	1.19	Prenol lipids
pos_4643	4,4-Dimethyl-5alpha-cholesta-8-en-3beta-ol	6.89	0.031	1.12	Steroids and steroid derivatives
pos_2647	CDP-DG(a-25:0/20:5(5Z,8Z,11Z,14Z,16E)-OH(18R))	5.10	<0.001	1.19	Glycerophospholipids
neg_2949	Glycyl-Serine	4.35	<0.001	1.19	Carboxylic acids and derivatives
neg_3119	CDP-DG(a-21:0/PGF1alpha)	3.97	<0.001	1.19	Glycerophospholipids
pos_1406	2′,3′-Dideoxyuridine	3.51	0.002	1.15	Pyrimidine nucleosides
pos_1375	Lumiflavin	3.48	0.002	1.17	Pteridines and derivatives
neg_4018	28-Glucosyl-19(29)-dehydroursolic acid 3-arabinoside	3.45	<0.001	1.19	Prenol lipids
neg_3398	Maslinic acid 3-O-b-D-glucoside	3.23	<0.001	1.19	Prenol lipids
neg_4630	DG(15:0/20:3(5Z,8Z,11Z)-O(14R,15S)/0:0)	3.02	0.008	1.16	Glycerolipids
neg_2649	Erythromycin D	2.80	<0.001	1.19	Peptidomimetics
pos_2144	Didemnin A	2.60	<0.001	1.19	Peptidomimetics
pos_1543	Deoxyloganic acid	2.60	<0.001	1.19	Prenol lipids
Down-regulated					
pos_4065	Methymycin	−7.38	0.045	1.10	Macrolides and polyketides
pos_4984	DG(14:1(9Z)/18:0/0:0)	−7.34	0.003	1.19	Glycerolipids
pos_4194	(-)-Jasmonic acid	−6.72	0.001	1.19	Fatty Acyls
pos_1297	Cotinine N-oxide	−5.82	0.001	1.19	Organoheterocyclic compounds
pos_1087	Clitocine	−3.65	<0.001	1.19	Pyrimidine nucleosides
pos_1934	p-Hydroxyphenobarbital	−3.45	<0.001	1.18	Organoheterocyclic compounds
pos_3145	1-(2-Furyl)butan-3-one	−3.42	0.003	1.16	Organoheterocyclic compounds
pos_4341	2-[[(2S)-2-acetamido-4-methylpentanoyl]amino]-N-[5-(diaminomethylideneamino)-1-oxopentan-2-yl]-4-methylpentanamide	−3.42	0.011	1.16	Carboxylic acids and derivatives
neg_4523	Cortol	−3.15	0.003	1.17	Steroids and steroid derivatives
pos_4350	Erinapyrone A	−2.90	0.003	1.14	Organoheterocyclic compounds
pos_4529	5-Phenyl-1,3-pentadiyne	−2.74	0.026	1.12	Benzene and substituted derivatives
neg_2309	1-Methylguanosine	−2.67	0.023	1.13	Purine nucleosides
pos_3978	PGD2 ethanolamide	−2.41	<0.001	1.19	Fatty Acyls
neg_2002	Ret-P-man	−2.40	<0.001	1.19	Carbohydrates and carbohydrate conjugates
neg_2303	Comuside	−2.34	<0.001	1.19	Prenol lipids

## Data Availability

The RNA-seq data have been deposited in the Sequence Read Archives (SRA) under accession number PRJNA1481800 at the National Center for Biotechnology Information (NCBI). The metabolomics data have been deposited in the Science Data Bank (ScienceDB) and are accessible at the following link: https://doi.org/10.57760/sciencedb.41966.

## Supplementary Materials

The following supporting information can be downloaded at: https://www.mdpi.com/article/10.3390/insects17080827/s1, Table S1: Primers used in RT-qPCR validation; Table S2: Summary of differentially expressed genes (DEGs); Table S3: Summary of significant and non-significant differential metabolites; Table S4: Integrated gene–metabolite correlation analysis; Figure S1: PCA analysis of all samples; Figure S2: Assessment of replicate correlations. (A) Correlation plot among samples; (B) correlation plot among QC samples; Figure S3: Orthogonal partial least squares discriminant analysis (OPLS-DA). (A) OPLS-DA score plot; (B) OPLS-DA scatter plot; Figure S4: Functional annotation and classification of differentially expressed genes (DEGs) in *Liriomyza trifolii*. (A) COG functional classification; (B) GO functional classification; Figure S5: RT-qPCR validation of differentially expressed genes identified by transcriptomic analysis. (A) Means (±SE) were used to evaluate expression with the 2^−ΔΔCt^ method. Relative mRNA expression is shown on the left y-axis, and log-transformed FPKM values are shown on the right y-axis. (B) Linear correlation analysis of qPCR and RNA-seq data.
