# Supplementary material for: Bioactivity and Molecular Responses of Bitter Gourd Leaf Extracts Against the Invasive Leafminer, Liriomyza trifolii (Diptera: Agromyzidae)
Source: Insects. 2026 Aug 10;17(8):827. doi: 10.3390/insects17080827 (PMC13513763; doi:10.3390/insects17080827)
Supplement: Supplementary file 1 [file insects-17-00827-s001.zip › Supplementary material.pdf]

Supplementary material

Table S1 Primers used in RT-qPCR validation

Gene		Primer sequences (5'→3')
Ltr01G000530.1	Forward	GCCGCTTTCTTTGGACCTC
	Reverse	TGCCATCTTCATTCACATTCG
Ltr01G001030.1	Forward	CGTTCAATACGCCCAGACAA
	Reverse	CTGAGCCACCTGCTGACAAT
Ltr05G005520.1	Forward	AACATACCCTTGGACTTTCGTG
	Reverse	ATGGCCTGCTTGACCTGAG
Ltr05G009230.1	Forward	AAGTATGCCGAGAAAGCAAAAG
	Reverse	TGCAACATGCTCCACCCAC
Ltr05G008090.1	Forward	TTCGGAAGCCTTGAAACCA
	Reverse	TCATTGCCACTCACCCACATA
Ltr01G012840.1	Forward	CACAAACAATCAAGCACGGAA
	Reverse	CAGCACTGACCAATTTAGACGC
LtrUnG013390.1	Forward	TCAGAAGGGCATACGAGGATT
	Reverse	CGGGGCGTATAACTATGGGTT
NewGene_192.1	Forward	ATGAGGCGGAGGGTGATGA
	Reverse	ATTGCGGAAGATTCAGTTGGT

Table S2 Summary of differentially expressed genes (DEGs).

Table S3 Summary of significant and non-significant differential metabolites.

Table S4 Integrated gene–metabolite correlation analysis.

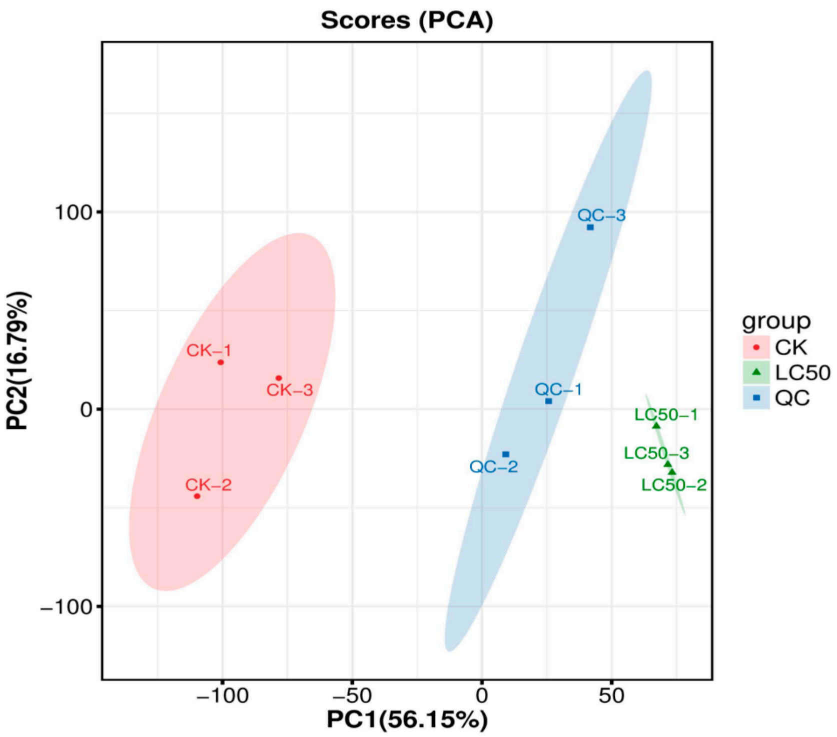

Figure S1. PCA analysis of all samples.

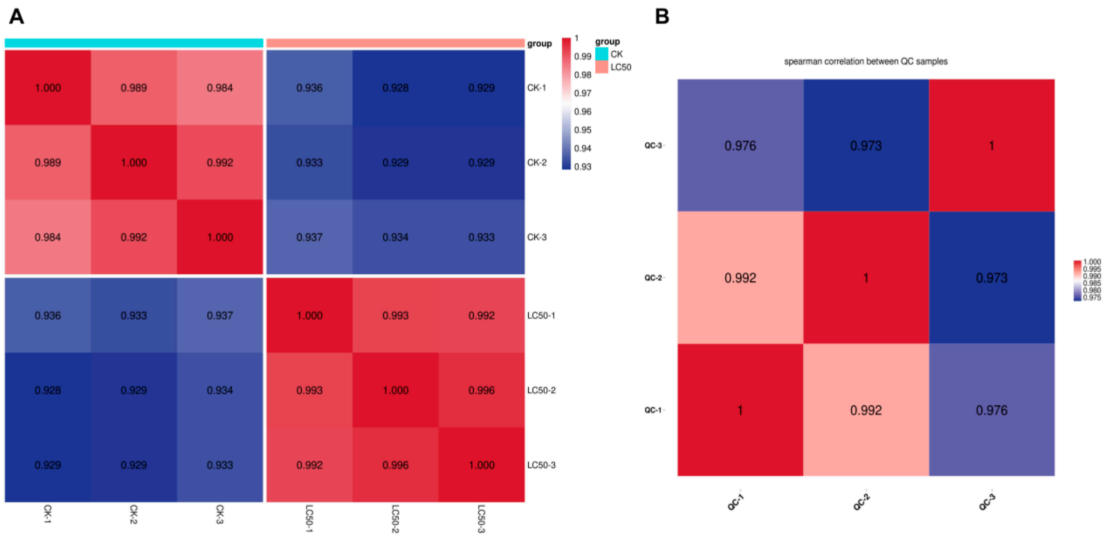

Figure S2. Assessment of replicate correlations. (A) Correlation plot among samples; (B) correlation plot among QC samples.

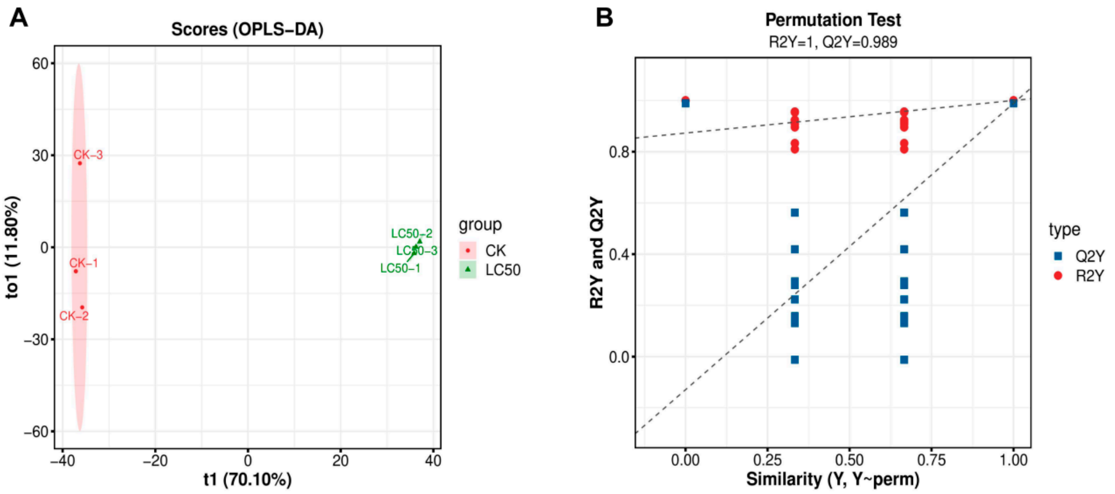

Figure S3. Orthogonal partial least squares discriminant analysis (OPLS-DA). (A) OPLS-DA score plot; (B) OPLS-DA scatter plot.

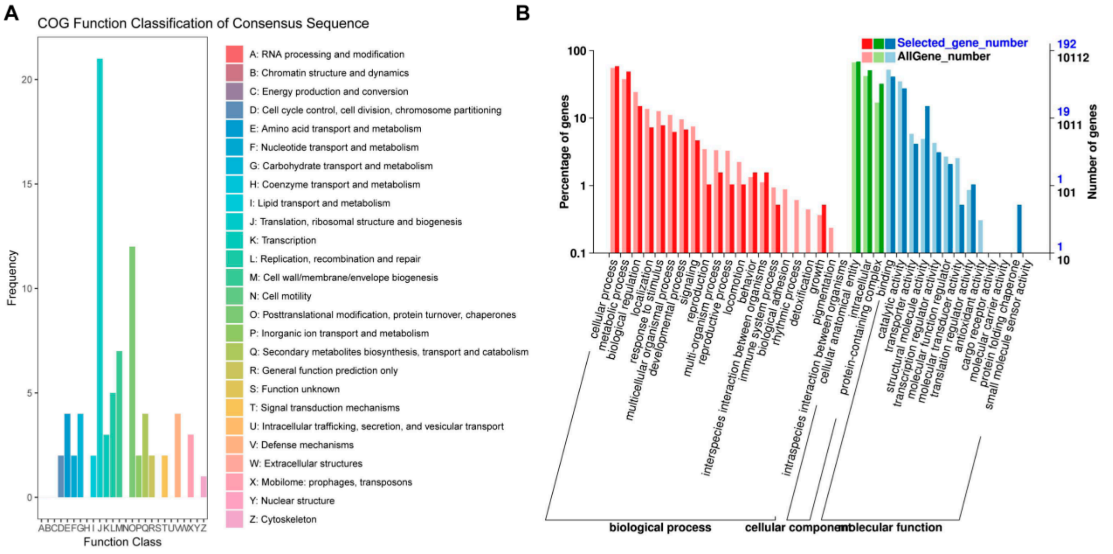

Figure S4. Functional annotation and classification of differentially expressed genes (DEGs) in *Liriomyza trifolii*. (A) COG functional classification; (B) GO functional classification.

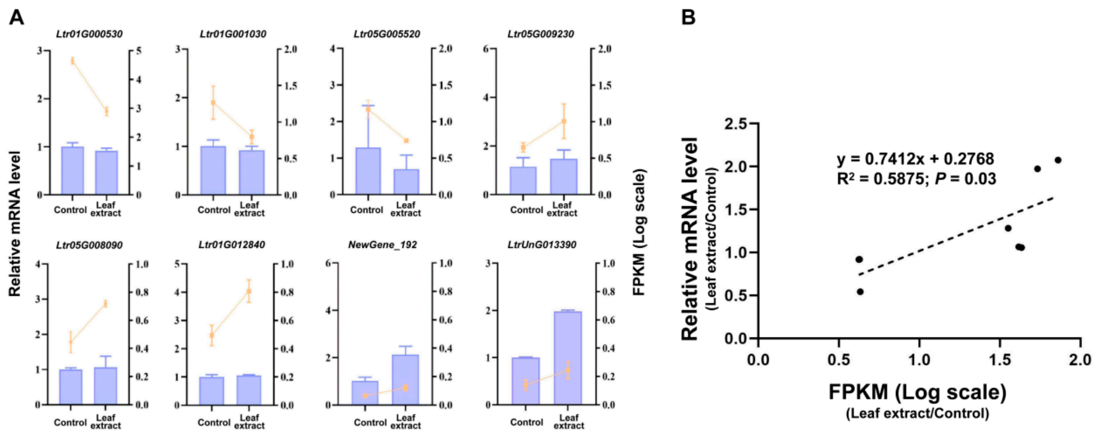

Figure S5. RT-qPCR validation of differentially expressed genes identified by transcriptomic analysis. (A) Means (±SE) were used to evaluate expression with the $2^{-\Delta\Delta Ct}$ method. Relative mRNA expression is shown on the left y-axis, and log-transformed FPKM values are shown on the right y-axis. (B) Linear correlation analysis of qPCR and RNA-seq data.
