# Supplementary material for: Bioactivity and Molecular Responses of Bitter Gourd Leaf Extracts Against the Invasive Leafminer, Liriomyza trifolii (Diptera: Agromyzidae)
Source: Insects. 2026 Aug 10;17(8):827. doi: 10.3390/insects17080827 (PMC13513763; doi:10.3390/insects17080827)
Supplement: Supplementary file 1 [file insects-17-00827-s001.zip › Table S1 Primers used in RT-qPCR validation.pdf]

Table S1 Primers used in RT-qPCR validation.

Gene		Primer sequences (5'→3')
Ltr01G000530.1	Forward	GCCGCTTTCTTTGGACCTC
	Reverse	TGCCATCTTCATTCACATTCG
Ltr01G001030.1	Forward	CGTTCAATACGCCCAGACAA
	Reverse	CTGAGCCACCTGCTGACAAT
Ltr05G005520.1	Forward	AACATACCCTTGGACTTTCGTG
	Reverse	ATGGCCTGCTTGACCTGAG
Ltr05G009230.1	Forward	AAGTATGCCGAGAAAGCAAAAG
	Reverse	TGCAACATGCTCCACCCAC
Ltr05G008090.1	Forward	TTCGGAAGCCTTGAAACCA
	Reverse	TCATTGCCACTCACCCACATA
Ltr01G012840.1	Forward	CACAAACAATCAAGCACGGAA
	Reverse	CAGCACTGACCAATTTAGACGC
LtrUnG013390.1	Forward	TCAGAAGGGCATACGAGGATT
	Reverse	CGGGGCGTATAACTATGGGTT
NewGene_192.1	Forward	ATGAGGCGGAGGGTGATGA
	Reverse	ATTGCGGAAGATTCAGTTGGT
